# Case Report: Expanding the phenotypic spectrum of PURA syndrome in South America with the first presentation of concurrent vitiligo

**DOI:** 10.3389/fped.2024.1323014

**Published:** 2024-03-28

**Authors:** S. Mora-Martinez, Natalia Castaño-Giraldo, Humberto Alejandro Nati-Castillo, Laura Barahona Machado, Tatiana Mora Arbeláez, G. Gordillo-Gonzalez, Juan S. Izquierdo-Condoy

**Affiliations:** ^1^ Corporación Universitaria Empresarial Alexander von Humboldt, Armenia, Colombia; ^2^ Faculty of Health Sciences, Universidad Alexander von Humboldt, Armenia, Colombia; ^3^ Department of Internal Medicine, Interinstitutional Internal Medicine Group (GIMI 1), Universidad Libre, Cali, Colombia; ^4^ Faculty of Health Sciences, Universidad del Quindío, Armenia, Colombia; ^5^ Clinical Genetics Department, Universidad Cooperativa de Colombia, Santa Marta, Colombia; ^6^ One Health Research Group, Universidad de las Américas, Quito, Ecuador

**Keywords:** PURA syndrome, genetic, intellectual disability, diagnosis barriers, South America

## Abstract

Purine-rich element-binding protein A (PURα) regulates multiple cellular processes. Rare *de novo* mutations can lead to PURA syndrome, which manifests as a range of multisystem disturbances, including hypotonia, global developmental delay, swallowing disorders, apnea, seizures, visual impairments, and congenital heart defects. We report the case of a Colombian girl with no relevant medical history who was diagnosed with PURA syndrome at the age of 7, due to a heterozygous mutation located at 5q31.2, specifically the variant c.697_699del (p.Phe233del), in exon 1 of the PURA gene. This represents the first documented case of PURA syndrome in South America and the first association of the syndrome with vitiligo, thereby expanding the known phenotypic spectrum. In addition to enriching the literature concerning the phenotypic diversity of PURA syndrome, this report highlights, for the first time, the diagnostic challenges faced by developing countries like Colombia in diagnosing high-burden rare diseases such as PURA syndrome.

## 1 Introduction

Purine-rich element binding protein A (PURα) plays a crucial role in postnatal neurodevelopment and is implicated in processes such as neuronal proliferation, dendritic maturation, myelination, and the transport of mRNA to translation sites across multiple tissues (1). Since 2014, heterozygous mutations in the gene encoding PURα have been linked to a variety of neurodevelopmental disorders (2). PURA syndrome commonly manifests during the neonatal stage and exhibits a diverse range of clinical symptoms, including but not limited to hypotonia, respiratory complications, swallowing disorders, drowsiness, hypothermia, and intellectual disability. Consequently, developmental milestones, including head control, rolling over, sitting, crawling, and walking, are often delayed in individuals with the syndrome (3). Additionally, the syndrome is frequently accompanied by growth retardation and is also associated with epilepsy, gastrointestinal issues, endocrine disorders, and ophthalmological abnormalities (4, 5).

Vitiligo, the most prevalent skin pigmentation disorder globally, is characterized by hypomelanosis, which manifests as hypochromic or achromic macules (6, 7). The pathophysiology of vitiligo is intricate, with the predominant theory attributing the loss of functional melanocytes to an autoimmune response. This is thought to be associated with alterations in genes involved in both innate and adaptive immune systems (8, 9).

Despite considerable advances in molecular genetics, the complete clinical characterization and epidemiological profile of PURA syndrome remain inadequately studied. Notably, no cases from South America have been reported in the existing scientific literature, leaving a significant gap in our understanding of the syndrome's geographical prevalence and its distribution among various ethnic groups (10–13). In light of this, we present the first documented case of PURA syndrome in South America, focusing on a pediatric patient from Colombia who received a delayed diagnosis at the age of seven. The diagnosis was confirmed through next-generation whole exome sequencing, revealing a heterozygous variant, specifically c.697_699del (p.Phe233del), in exon 1 of the PURA gene. Interestingly, the clinical presentation of this case was further complicated by the concurrent manifestation of vitiligo.

## 2 Case description

The patient is a seven-year-old female born in Quimbaya, a city within the municipality of Quindío, Colombia. She has no significant family medical history of either PURA Syndrome or vitiligo. Despite an unremarkable prenatal history and normal physical attributes at birth, her clinical presentation gradually became more complicated as she grew older.

**Figure 1:**
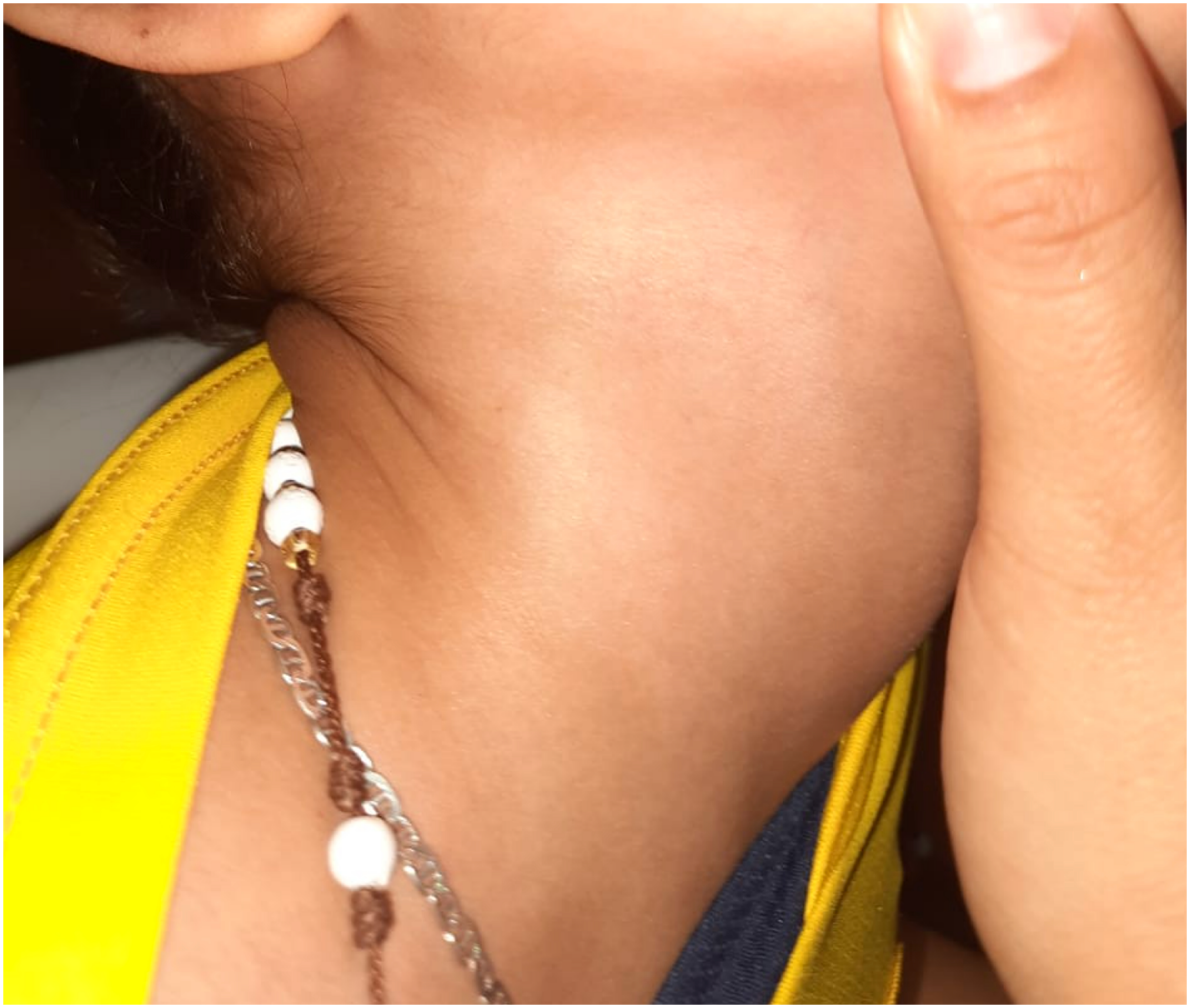
Hypopigmented macula: diagnosed as vitiligo by dermatology specialist.

**Figure 2:**
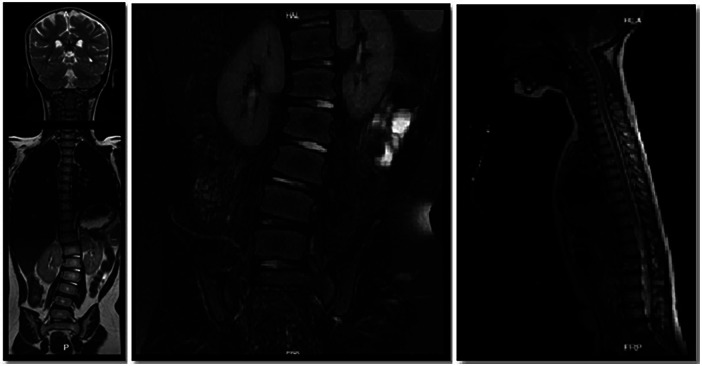
MRI of the spine: the presence of the lumbosacral transition vertebra as the sixth lumbar vertebra is evidenced. There is a right rotoscoliosis with a 22° Cobb angle between L1 and L4. With rectification of the physiological curve of the spine in the dorsal and lumbar region and inversion of the cervical lordotic curve.

Eight months postpartum, she manifested generalized hypotonia, lack of head control, dermatological irregularities, frequent hiccups, and constipation. The intricacies of her symptoms necessitated multiple specialist evaluations. A neuropediatric assessment identified global developmental delays, including walking delay within developmental milestones, and dysphagia. While a dermatological examination revealed a single hypopigmented macule in the right cervical region, resulting in a diagnosis of segmental-unisegmental vitiligo (Figure 1). Orthopedic evaluation further complicated the clinical picture with findings of a right hip dislocation, subluxation of the right coxofemoral joint, and left coxa valga; surgical interventions were required for these conditions. Additionally, imaging studies were equally complex; contrast-enhanced computed tomography (CT) scans of the head indicated cortical atrophy and plagiocephaly, and lumbar scoliosis with a left convexity of 22 degrees between L1 and L5, and thoracic scoliosis with a right convexity of 20 degrees between T6 and T12, were observed in radiography and magnetic resonance imaging of the spine (Figure 2).

**Figure 3:**
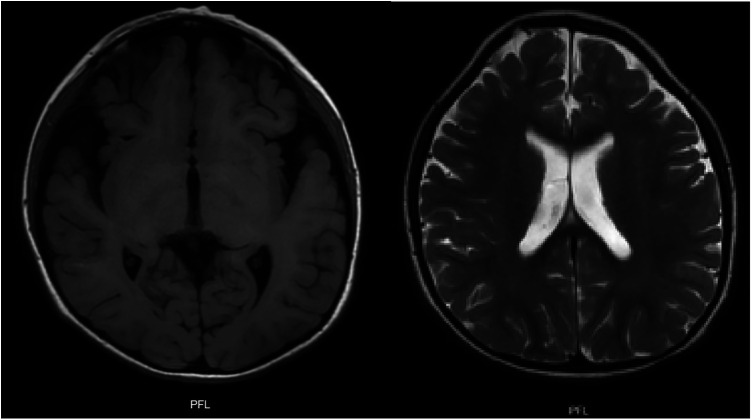
T1 and T2 weighted brain MRI: plagiocephaly, cortical atrophy, involvement of the white matter and frontal lobes is observed. Generalized increase in the amplitude of the subarachnoid space and ventricular system.

The multi-systemic nature of her symptoms and the need for diverse specialist evaluations elongated the diagnostic journey. By the age of four, persistent neurodevelopmental delays were accompanied by constipation and feeding difficulties. Additional advanced investigations were conducted to pinpoint a diagnosis. Brain magnetic resonance imaging (MRI) disclosed progressive abnormalities involving the frontal lobes, as well as an expanded subarachnoid space and ventricular system (Figure 3). Esophageal manometry verified swallowing disorders, while polysomnography identified obstructive hypopneas, resulting in a diagnosis of moderate obstructive sleep apnea-hypopnea syndrome (OSAHS) (apnea-hypopnea index of 11.9); this was managed with continuous positive airway pressure (CPAP) therapy. Table 1 chronologically summarizes the analysis performed on the patient.

**Table 1:** Timeline of paraclinical examinations.

Paraclinical date	Patient age	Result
24/10/2014	8 months	Brain computed tomography scan: Cortical atrophy and plagiocephaly.
13/01/2016	1 year and 10 months	Total CPK: 101.5; ammonium: 21; lactic acid: 15.7; Qualitative amino acids in plasma and urine: normal.
23/01/2016	1 year 11 months	Brain nuclear magnetic resonance: decreased volumes of the frontal lobes with a symmetrical appearance with a prefrontal predominance associated with a reduction in the volume of the skull vault; auditory evoked potentials: normal.
15/02/2016	1 year 11 months	Panoramic spine x-ray: scoliosis in the thoracic spine.
22/02/2016	1 year 11 months	Karyotype: 46, XX [16].
23/02/2016	1 year 12 months	Abdominal ultrasound and echocardiogram: normal.
21/01/2018	3 years 10 months	Brain nuclear magnetic resonance: delayed myelination, mild ventriculomegaly, increased subarachnoid space; Gaucher screening: negative; lactate, pyruvate, and ammonium in blood: normal.
20/09/2018	4 years 6 months	Hip x-ray: dysplastic changes of the left hip.
23/10/2018	4 years 8 months	Electroencephalogram: without epileptiform activity or alteration in background rhythms. Echocardiogram: normal.
06/11/2018	4 years 8 months	CLN1 and CLN2: normal enzyme activity. High Resolution Comparative Genomic Hybridization: normal.
04/12/2018	4 years 9 months	Polysomnography: severe sleep apnea; beta glucocerebrosidase activity: 6.8 uM.
16/01/2020	5 years 11 months	TSH: 1.58 mUI/L; VDRL: not reactive; ANAS: negative; MLPS 15q11q13: no deletions or duplications, or alteration of methylation.
27/11/2020	6 years 9 months	Exome sequencing: pathogenic variant in heterozygous state: c.697_699del (p.Phe233del), in exon 1 of the PURA gene.
23/07/2021	7 years 5 months	Somatomedin C: 169 ng/ml; HbA1c: 4.69% Cortisol: 9.29 mcg/dl; TSH 1.79 mUI/L; FT4: 0.86 ng/dl.
14/11/2021	7 years 8 months	Hip x-ray: Left hip fracture in the process of consolidation with osteosynthesis material *in situ*.

CPK, creatine phosphokinase; CLN, neuronal ceroid lipofuscinoses; HbA1c, glycated hemoglobin; TSH, thyroid stimulating hormone; FT4, free thyroxine hormone.

## 3 Diagnosis and treatment

The diagnostic journey for this patient was notably intricate, owing to her polysystemic symptoms. CLN1 and CLN2 enzyme activity was normal, eliminating the possibility of Batten disease. Initial genetic screening targeted suspected Angelman syndrome and utilized high-resolution comparative genomic hybridization, along with multiplex ligation-dependent probe amplification (MLPA). Both tests yielded normal results, thereby extending the timeframe needed for securing a definitive diagnosis. In light of this prolonged uncertainty, whole-exome sequencing was eventually performed. Chromosomal microarray detected a heterozygous mutation located at 5q31.2, and the whole exome sequencing based on 20× depth of coverage percentage (99.8%) revealed a specifically c.697_699del (p.Phe233del), which confirmed the diagnosis of PURA syndrome when the patient was 7 years old.

Upon confirming the diagnosis of PURA syndrome, the treatment strategy was re-evaluated and tailored to meet the patient's specific needs. Antiepileptic medication, specifically carbamazepine, was prescribed for non-motor seizures, altered states of consciousness, and abnormal findings on video telemetry. In addition, a comprehensive rehabilitation program was initiated, which included daily physical therapy sessions to enhance her compromised motor skills and address her inability to walk independently. The program was based on tactile stimulation, vestibular and proprioceptive stimulation, as well as the implementation of seated and bipedal straightening reactions with the reeducation of postural patterns, muscle strengthening, and maintenance of joint ranges with arcs of motion. The implemented therapies have been closely monitored for a period of one year, during which the patient has demonstrated stable and sustained improvement, particularly in neurological and motor symptoms.

## 4 Discussion

This report introduces for the first time a pediatric patient from Colombia—and seemingly the first in South America—with a variety of non-specific multisystem alterations. These were ultimately attributed to PURA Syndrome and were found in conjunction with segmental–unisegmental vitiligo (14, 15). Globally, the incidence of PURA Syndrome is exceptionally rare, primarily being diagnosed in newborns. As documented by the Pura Syndrome Foundation, only 478 cases had been reported globally as of 2021, and few have been added to the existing literature thereafter (11, 13).

The specific variant of PURA identified in this case, p.(Phe233del), was first described in the United Kingdom in 2014 (2). Subsequent case series scans have revealed a significant frequency of drug-resistant epilepsy and cardiac abnormalities in patients with this variant (4). Currently, it is considered the most frequent variant (16). However, this case report thus marks the first occurrence of this heterozygous PURA mutation variant c.697_699del (p.Phe233del) in Colombia. Uniquely, it is also the first instance where this variant has co-occurred with vitiligo.

The case described here warrants particular attention due to its complex clinical profile and the late age of diagnosis—seven years old (17). Additionally, although genetic screening is indicated, the patient's parents did not have access to these tests. This serves to highlight the ongoing challenges faced in the timely recognition of PURA Syndrome in developing countries like Colombia, where healthcare professionals must often rely on a high level of clinical suspicion over a prolonged period, owing to limited access to molecular diagnostic tests.

Despite the concomitant presentation of PURA syndrome with vitiligo, we consider that the multifactorial origin of vitiligo suggests that this confluence is primarily an incidental finding (6). Nevertheless, we believe that this report opens the door to the possibility that PURA Syndrome might be associated with autoimmune diseases such as vitiligo. Typically, symptoms of PURA Syndrome appear immediately post-birth and affect the early stages of development (12). The patient in this study presented with a delay in neurodevelopment and exhibited pronounced language impairment, while also lacking the ability to walk or communicate verbally independently.

Given the extreme rarity of this condition, the clinical and phenotypic characterization of PURA Syndrome remains a subject of ongoing research and debate (13). Most cases involving heterozygous mutations in the PURA gene, like this one, are characterized by symptoms such as hypotonia (97%), resulting in moderate to severe global developmental delay and issues related to swallowing disorders (81%) (18, 19). Additionally, PURA Syndrome has been associated with less frequent symptoms like apnea, seizures or epilepsy (50%), visual impairment, congenital heart defects, urogenital malformations, skeletal anomalies, and endocrine disorders (4, 20).

**Figure 4:**
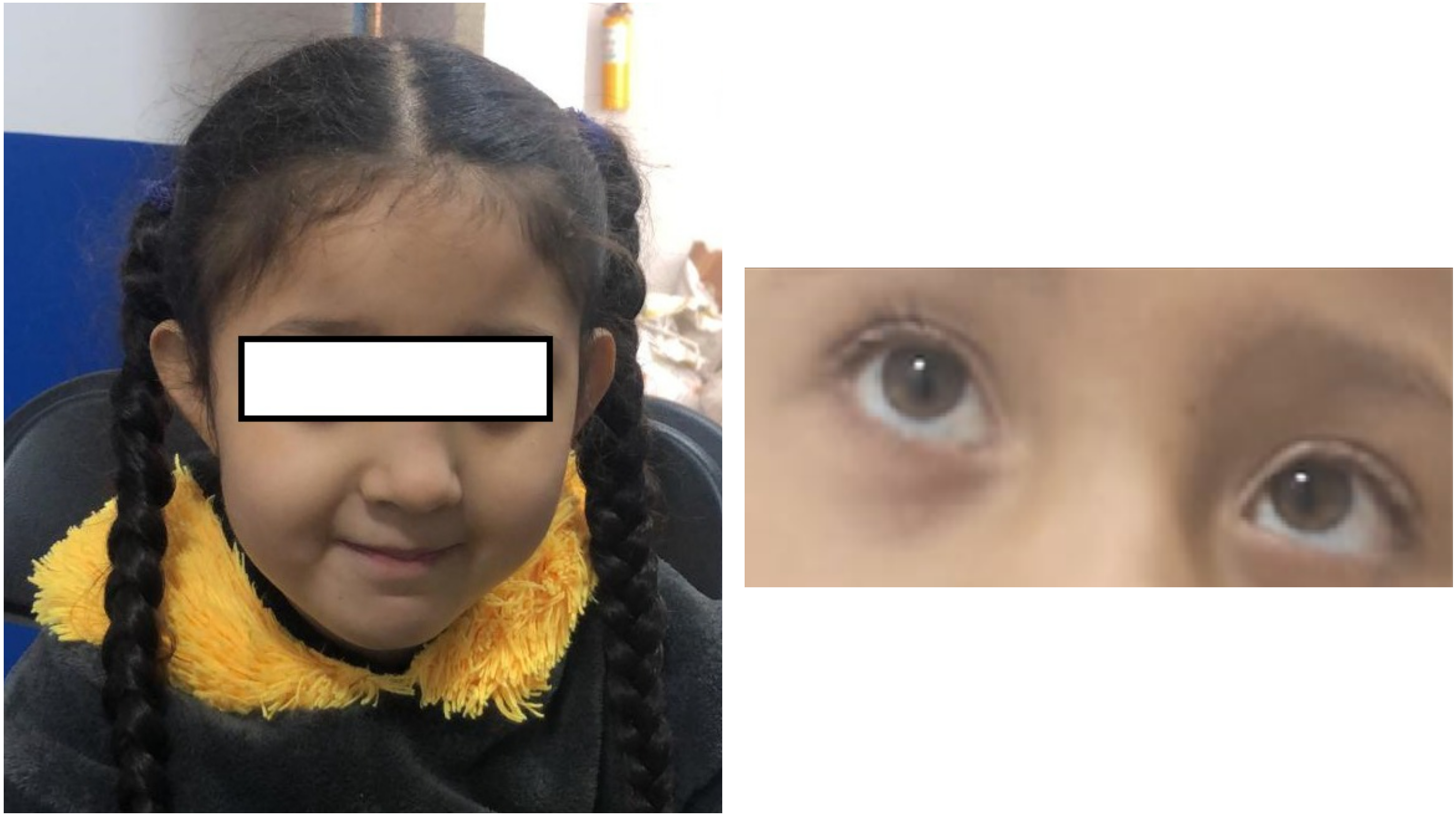
Phenotype: no characteristic dimorphism is seen in other patients with PURA syndrome, despite the presence of plagiocephaly and convergent left strabismus.

Our patient presented with a complex clinical picture, including generalized hypotonia, constipation, frequent hiccups, dysphagia, a hypopigmented macular lesion in the right cervical region, and compromised hip mobility. Although facial dysmorphism may accompany PURA syndrome, it was not evident in this patient. However, the patient had plagiocephaly and left convergent strabismus (Figure 4), with the latter being a frequently described feature among patients with PURA syndrome (19, 21).

In terms of neuroimaging findings, microdeletions at the 5q31.3 locus are known to impact the brain, affecting both white matter and the frontal lobes (22). Initially, these alterations are latent but progress over time to result in volume loss, delayed myelination, and demyelination. In line with this, the patient's initial brain MRI displayed a reduction in the frontal lobe volume, a finding corroborated by similar cases (23, 24).

Currently, there is no specific treatment for PURA Syndrome; care is generally symptomatic, with rehabilitation therapy as the primary form of intervention. This therapy should be tailored to the patient's requirements, and its correct and timely application can yield positive results, as seen in this patient.

This case significantly expands the phenotypic spectrum linked to PURA mutations and offers new insights into the clinical manifestations specific to the South American population. It also underscores the difficulties faced by patients with rare disorders like PURA Syndrome in developing countries such as Colombia. The scarcity of diagnosed cases suggests that exome sequencing may help identify more individuals affected by this condition (17). This state of affairs emphasizes the need for increased support from state health organizations and the private sector, including pharmaceutical companies, especially for patients grappling with very rare diseases that impose a significant burden on both the individuals and their families (25).

## 5 Patient perspective

From the caregivers' perspective, navigating an undiagnosed condition from a young age was both confusing and isolating. The journey to a diagnosis involved numerous tests and visits to specialists. Each visit came with its own set of challenges and unanswered questions, further complicated by a lack of resources. The complexity of the symptoms made daily activities discouraging.

Receiving a diagnosis of PURA syndrome at the age of 7 brought both relief and new challenges for the caregivers. It helped clarify the medical uncertainties but also emphasized the rarity of the condition and the intricacies of its management. The multifaceted treatment plan, which includes medication and physical therapy, has improved the patient's quality of life, particularly in addressing neurological and motor symptoms.

## 6 Conclusions

The authors report the particularities and barriers associated with the first case of PURA Syndrome in Colombia, which is secondary to the heterozygous variant c.697_699del (p.Phe233del).

The complex array of manifestations for PURA Syndrome, coupled with the necessity for exome sequencing to confirm its diagnosis, complicates its identification, particularly in developing nations like Colombia.

Signs such as hypotonia, global developmental delay, intellectual disability, and multisystem manifestations should raise suspicion of this syndrome.

Imaging analyses, especially brain MRIs, can be instrumental in documenting frontal lobe volume loss, demyelination, ventriculomegaly, and other manifestations.

PURA Syndrome is a recently described pathology; hence, the publication of new case reports and studies is essential for improving our understanding of this ultra-orphan disease, especially in the South American region.

## Data Availability

The original contributions presented in the study are included in the article/Supplementary Material, further inquiries can be directed to the corresponding author.
